# Functionalization of Two-Component Gelatinous Peptide/Reactive Oligomer Hydrogels with Small Molecular Amines for Enhanced Cellular Interaction

**DOI:** 10.3390/ijms26115316

**Published:** 2025-05-31

**Authors:** Caroline Kohn-Polster, Benno M. Müller, Jan Krieghoff, Awais Nawaz, Iram Maqsood, Annett Starke, Kirsten Haastert-Talini, Michaela Schulz-Siegmund, Michael Christian Hacker

**Affiliations:** 1 Pharmaceutical Technology, Institute of Pharmacy, Medical Faculty, Leipzig University, Eilenburger Straße 15a, 04317 Leipzig, Germanyjan.krieghoff@uni-leipzig.de (J.K.); awais@uvas.edu.pk (A.N.); irammaqsood2@yahoo.com (I.M.); annett.starke@uni-leipzig.de (A.S.); michaela.schulz-siegmund@medizin.uni-leipzig.de (M.S.-S.); 2 Institute of Pharmaceutical Sciences, University of Veterinary & Animal Sciences (UVAS), Outfall Road, Lahore 54000, Pakistan; 3 School of Pharmacy, University of Maryland, 20 North Pine Street, Baltimore, MD 21201, USA; 4 Hannover Medical School, Institute of Neuroanatomy and Cell Biology, Carl-Neuberg-Straße 1, 30625 Hannover, Germany; haastert-talini.kirsten@mh-hannover.de; 5 Center for Systems Neuroscience (ZSN) Hannover, Bünteweg 2, 30559 Hannover, Germany; 6 Faculty of Mathematics and Natural Sciences, Institute of Pharmaceutics and Biopharmaceutics, Heinrich Heine University Düsseldorf, Universitätsstraße 1, 40225 Düsseldorf, Germany

**Keywords:** in situ-forming hydrogels, oligomeric cross-linkers, chemical modification, gelatin, adipose-derived stem cells, Schwann cells, nerve regeneration

## Abstract

A platform of two-component cross-linked hydrogel (cGEL) based on gelatinous peptides and anhydride-containing cross-linkers (oPNMA, oPDMA) is extended for use in peripheral nerve regeneration. Hybrid composites with bio-/chemical cues for enhanced biophysical and biochemical properties were fabricated by covalently grafting small molecular, heterobifunctional amines including the nerve growth factor mimetic LM11A-31 to the oligomeric cross-linkers prior to hydrogel formation. The cytocompatibility and growth-supportive conditions within the matrix are confirmed for pristine and modified hydrogels using L929 mouse fibroblasts and human adipose-derived stem cells (hASCs). For hASCs, cell behavior depends on the type of cross-linker and integrated amine. In a subsequent step, neonatal rat Schwann cells (SCs) are seeded on pristine and functionalized cGEL to investigate the materials’ capabilities to support SC growth and morphology. Within all formulations, cell viability, adherence, and cell extension are maintained though the cell elongation and orientation vary compared to the two-dimensional control. It is possible to merge adjustable two-component hydrogels with amines as biochemical signals, leading to improved nervous cell proliferation and activity. This indicates the potential of tunable bioactive cGEL as biomaterials in nerve implants, suggesting their use as a foundational component for nerve conduits.

## 1. Introduction

In regenerative medicine, hydrogel materials are of high interest for biomedical applications such as cell therapy or tissue engineering. Based on various natural or synthetic composites and cross-linking strategies, numerous two- (2D) and three-dimensional (3D) hydrogel systems have been developed [1,2,3]. With regard to stem cell-based therapy, hydrogels constitute a specialized microenvironment as they mimic native extracellular matrix (ECM). The ECM represents a niche or stem cell regulator with local interactions (e.g., biophysical, biochemical) and architectural limitations allowing for tissue signals and feedback cascades [1,4]. Due to the link between the cytoskeleton and ECM, unique cellular responses and mechanosensing of the surrounding matrix are possible. For example, the creation of contractile forces is seen via focal-adhesion anchored stress fibers that act as force-generating transducers of the matrix rigidity [5,6,7]. These cell-material interactions depend on the microenvironment and matrix surface (e.g., biophysical and biochemical cues, cultivation conditions, degradability) and regulate the cellular response (e.g., proliferation, differentiation, cell fate). In reverse, cellular remodeling can change the material characteristics such as accessible mechanical cues [5,8]. During biomaterial/hydrogel development, the material properties should be adjusted to reach desired cell-substrate interactions. This accounts mainly for physical material properties (e.g., polymer crystallinity, surface topography, matrix stiffness) that are known to initiate specific cellular responses [6,9,10]. Exemplarily, the material stiffness induces an outside-in signaling resulting in alterations of the integrin-mediated cell phenotype, adhesion, viability, and cytoskeleton organization [11,12]. Considering chemical material properties, the cellular response is modulated by factors like hydrophilicity, charge, or functional groups. For example, the material surface chemistry and charge can result in ionic interactions leading to conformational changes of integrin binding domains that change the proteogenic connection between the ECM and the cytoskeleton [13]. Finally, biological properties also contribute to biomaterial performance on a large scale. For instance, it is known that ECM proteins, predominantly fibronectin or laminin, or short mimetic peptides with the peptide sequence arginine-glycine-aspartic acid (RGD) enhance cell adhesion [8,14]. According to these considerations, the design of biomaterial (e.g., hydrogel systems) has to account for a combination of biophysical, biochemical, and biological properties to yield the desired biological responses in vitro and in vivo. The aim of this study was to extend the established platform of dual-component hydrogel (cGEL) by chemical functionalization with small molecular amines and, for the first time, to examine the in vitro cell reaction to these hybrid materials towards biomedical use in peripheral nerve regeneration (PNR) [15,16]. As the current medical reconstruction techniques and procedures to cure peripheral nerve injuries are insufficient to restore nerve architecture and locomotive/sensory functionality, a novel generation of nerve guidance conduits is required. With this motivation, cGEL two-component hydrogel composites are an interesting base material for nerve implants as luminal filler material in nerve conduits as they can be optimized to the required mechanical properties and degradability and allow for convenient biofunctionalization. In past studies, primary amine residues of collagen-derived gelatinous peptides (Collagel^®^ (COL) or gelatin type 160 Bloom (G160)) were cross-linked with multifunctional anhydride-containing oligomeric cross-linkers, oligo(PEDAS-co-NiPAAm-co-MA) (oPNMA) or oligo(PEDAS-co-DAAm-co-MA) (oPDMA) to pristine cGEL matrices. For chemical modification, reactive anhydride groups in oPNMA and oPDMA were partially derivatized immediately before cross-linking (pre-derivatization). Thereby, defined quantities of small monovalent primary amines were covalently integrated into the oligomer. For example, the NGF-hairpin loop 1 mimetic LM11A-31 was chosen for integration as it is described to positively affect dorsal root ganglion neurons by supportive neurotrophic activity [17]. These hybrid cGEL systems were analyzed concerning physicochemical properties and tested in vitro with L929 mouse fibroblasts, human adipose-derived stem cells (hASCs) as well as neonatal rat Schwann cells (nSCs). In detail, the cell viability, proliferation, migration, and spreading in response to the hydrogels were monitored and the effect of the biofunctionalization with small heterobifunctional amine molecules was evaluated. Overall, this study yielded to extend the potential of functionalized cGEL for the perspective design of novel nerve guidance conduits.

## 2. Results and Discussion

The dual-component hydrogel system composed of gelatinous peptides (COL, G160) and anhydride-containing macromer building blocks (oPNMA, oPDMA) was published before [15,18,19].

### 2.1. Oligomeric Building Blocks, Functionalization and Material Characterization of cGEL/cGEL(D) Matrices

**Figure 1:**
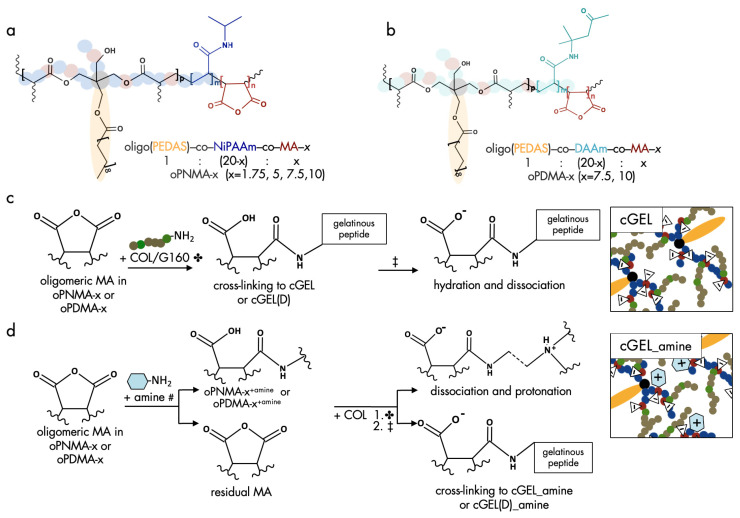
Outline of reactive building blocks and cross-linking with gelatinous peptides. (**a**) Oligomeric building block oligo(PEDAS-*co*-NiPAAm-*co*-MA) (oPNMA, left) with four different anhydride contents indicated by x, (**b**) block oligo(PEDAS-*co*-DAAm-*co*-MA) (oPDMA, right) with two different MA contents (x), semi-transparent schematic illustrations of oPNMA-x and oPDMA-x depict the branched structure of NiPAAm (blue)/DAAm (turquois) and MA (red) along the PEDAS core (black/yellow). (**c**) Cross-linking step ✤: oPNMA-x and oPDMA-x react with free amines in gelatinous peptides Collagel^®^ (COL) or gelatin (G160) under neutral pH to yield cross-linked cGEL and cGEL(D); ‡ washing and hydration in phosphate-buffered saline (PBS), anhydride cleavage and dissociation increase negative charge density. (**d**) Pre-derivatization #: covalent binding of 2.5 equivalents of intact anhydride groups in oPNMA-x or oPDMA-x to small molecular primary amines via amidation, formation of oPNMA-x^+amine^ or oPDMA-x^+amine^; cross-linking to cGEL_amine and cGEL(D)_amine: (1) ✤ residual anhydride group cross-linking with COL, (2) ‡ hydration in PBS leads to dissociation, residual anhydride cleavage and protonation of tertiary amines of incorporated amine. (**c**,**d**) Schematic illustrations of oligomers, primary amine, and COL in cGEL do not reflect actual dimensions (exemplarily shown for oPNMA); COL and G schematic: amino residues with primary amine (green bullet), any other amino acid (brown bullet); primary amine: residual aliphatic, aromatic, or heterocyclic compound (blue hexagon); triangles with minus marks depict negative charges, hexagons with positive marks depict partial cationic moieties.

For cross-linking of gelatinous peptides free amino acid residues are linked by zero-length or non-zero-length cross-linkers [20]. In the case of non-zero-length, cross-linkers (e.g., glutaraldehyde, genipin) are integrated into the hydrogel network. In this study, the cross-linker platform contained promising oligomers for non-zero-length cross-linking by providing multiple reaction sites. A graphical summary of the oligomeric cross-linkers oPNMA and oPDMA and the pre-derivatization of the oligomers are shown in Figure 1.

**Figure 2:**
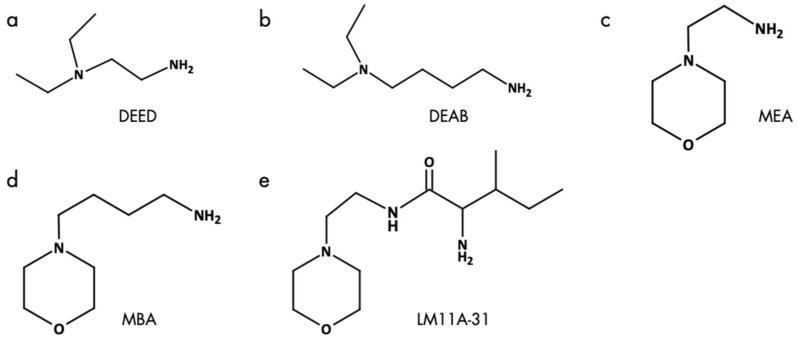
Amines for derivatization of oPNMA-x and oPDMA-x. (**a**) DEED: *N,N*-diethylethylendiamine, (**b**) DEAB: 4-(*N,N*-diethylamino)-butylamine, (**c**) MEA: 4-(2-aminoethyl)morpholine, (**d**) MBA: 4-(morpholin-4-yl)butan-1-amine, (**e**) LM11A-31: (2S,3S)-2-amino-3-methyl-*N*-[2-(4-morpholinyl)ethyl]pentanamide.

Based on the established synthesis of oPNMA and oPDMA, oligomeric building blocks with different anhydride contents were synthesized. The analysis of oPNMA-x and oPDMA-x batches showed that macromer properties including anhydride content and number-average molecular weight (M_n_) were in consensus with previous data for a range of different anhydride contents (refer to in Supplementary Materials Figure S1) [18,19]. Hence, batch-to-batch variations of the macromers did not bias resulting hydrogel properties. Upon cross-linking, primary amine groups in COL or G160 were covalently converted with anhydride groups in oPNMA or oPDMA to yield cGEL or cGEL(D), respectively (Figure 1). Under proton scavenging conditions (addition of the amine base NMPO), amide bonds were formed along with carboxylate moieties leading to a negative charge surplus in the hydrogel. In contrast to regular non-zero-length cross-linkers, oPNMA and oPDMA provide multiple reaction sites per oligomer chain leading to short and long-range cross-linking reactions. Thus, an effective conversion and substantial incorporation of the oligomeric building block was achieved. In addition to testing pristine dual-component hydrogels cGEL and cGEL(D), it was the aim of this study to improve the inherent hydrogel properties with (bio-)chemical functionalization. It was shown before that oPNMA can be pre-functionalized with primary amines prior to cross-linking to yield oPNMA-x^+amine^ (Figure 1c) [15,16,21]. In former work, oPNMA-x were derivatized with the fluorescent dye 6-aminoflourescein, and to generate a biological effect with the heterobifunctional diamine DEED and the NGF-mimetic LM11A-31 [15,16,18,21,22]. In the present study, additional amines, such as the butyl analog DEAB and the ethyl- and butylmorpholines MEA and MBA that were structurally inspired by the morpholine-derivative LM11A-31 were tested next to DEED (Figure 2).

In addition to a primary amine group, all candidates exhibited an additional non-nucleophilic tertiary amine. This bifunctional characteristic was chosen to enable covalent binding via the primary amine group and to provide a non-nucleophilic, basic structure for protonation within the matrix under physiological conditions. With this modification, cationic moieties and partial neutralization of the negative charge surplus were introduced to the hydrogel surface and bulk [19]. In preliminary work, it was seen that higher amine input in pre-derivatization resulted in steric constraints and increased amine protonation due to the reduced buffering capacity of the derivatized oligomer. Hence in this study, a low grafting quantity with MA equivalents of 2.5 was chosen. In the following, the cross-linking degree (CLD) of lyophilized hydrogels was determined as an indicator for material conversion during gelation (Table 1) [15].

In pristine cGEL matrices derived from oPNMA-x (x = 1.75, 5, 7.5, 10), the CLD values ranged between 89% to 92% and were constantly higher than reported before [15]. The same accounted for oPDMA-x (x = 7.5, 10) with CLD values above 80%. The comparison with previous data was limited due to varying gelation bases and concentrations, different fabrication techniques (full volume mixing, static mixing), and constitutions (disc, conduit). However, the high CLD emphasized that the hydrogel platform was flexible and feasible [15,16,19,21]. Regarding oPDMA-10-based hydrogels, the comparison of cGEL10 and cGEL(D)10 revealed a lower CLD. With the substitution of NiPAAm by DAAm, an additional methyl ketone functionality was introduced that possibly exacerbated the conversion of the MA functionalities with free amines in COL. This could be due to inaccessible anhydrides as well as fractional imine formation of amino residues in COL with ketones of oPDMA. For all novel hydrogel composites, cGEL10_amine and cGEL(D)10_amine, a high average CLD above 80% and 74% was determined, respectively. As the content of reactive MA and cross-linking ability was reduced by pre-derivatization with the heterobifunctional amines, these values ranged below corresponding pristine cGEL10 and cGEL(D)10. Nevertheless, the observed CLD indicated substantial material conversion and incorporation of the functionalized oligomers irrespective of the coupled amine. Regarding water content, hydrogels are generally characterized by a high water uptake that promotes tissue compatibility and diffusivity [20,23]. For cGEL, it was reported that the water content is inversely proportional to the concentration of gelatinous peptides and the number of covalent bonds within a certain range [15]. It can be assumed that incoherent hydrogels allow greater water influx and swelling of the network which are opposed by covalent cross-links and network retraction forces [24]. As another key parameter, the hydrogel stiffness was determined by oscillation rheology. From the different experimental setups and hydrogel specimens, a selection of representative storage moduli is depicted in Table 1. For oPNMA-x-derived hydrogels, storage moduli G’ were dependent on the peptide (e.g., COL or G160) and amine functionalization. In contrast to pristine cGEL10, cGEL10_amine gels showed lower storage moduli due to reduced CLD. For cGEL(D)10 and cGEL(D)_amine, the associated storage moduli G’ were less than half of the moduli for cGEL10 analogs. Like cGEL_amine matrices, the pre-derivatization of oPDMA-10 resulted in decreased hydrogel stiffness. However, a substantial impact of amine type and molecular size was not identified for cGEL(D) specimens. The material characterization illustrated that the choice of oligomeric cross-linker and type of pre-derivatization influenced cross-linking density, water content, and mechanical characteristics. It is well known that the hydrogel modulus critically influences cell adhesion, differentiation, migration, proliferation, and survival as each cell type is adjusted according to its native host tissue [25,26]. Therefore, it is claimed that softer materials mimic neurogenic tissue (i.e., brain) while increased stiffnesses comply better with myogenic and osteogenic tissue development [27].

In addition to the oligomeric cross-linker, the choice of gelatinous peptide significantly influences the biomaterial characteristics. Given the proteogenic nature and enzymatic degradability of COL, the cGEL and cGEL(D) hydrogel formulations presented here are likely to offer insufficient in vivo stability under certain conditions. These materials are designed to be fully biodegradable and resorbable through slow hydrolytic degradation, which can be accelerated by enzymatic activity depending on environmental conditions and cellular activity in close proximity to the gels. Preliminary studies demonstrated that the inclusion of collagenase A (0.1 U/mL in PBS) resulted in the complete degradation of cGEL discs within 60 h at body temperature. In contrast, cGEL conduits exhibited high hydrolytic stability in PBS for over six weeks but showed lower stability upon subcutaneous implantation in rats, lasting over two weeks. This highlights the enzymatic sensitivity of the cGEL material, particularly under in vivo conditions, and underscores the utility of cGEL/cGEL(D) as a degradable conduit filler material with bioactive molecules for enhanced peripheral nerve regeneration (PNR).

### 2.2. Cellular Response and Interaction of Fibroblasts and hASCs with cGEL and cGEL(D) Matrices

**Figure 3:**
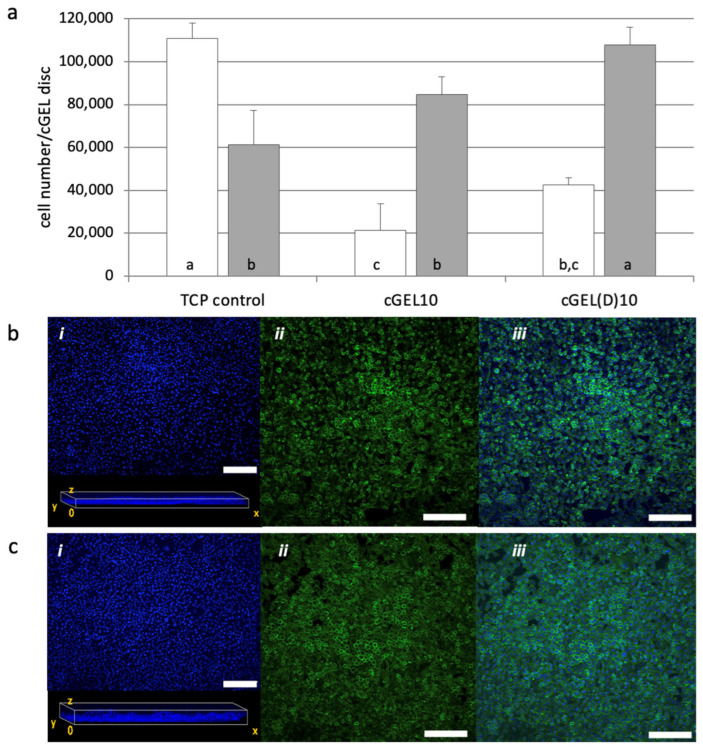
Direct cell contact of L929 mouse fibroblasts on cGEL10 and cGEL(D)10. (**a**) Number of fibroblasts on tissue culture plate (TCP) or hydrogel discs composed of COL cross-linked with oPNMA-10 (cGEL10) or oPDMA-10 (cGEL(D)10); cell number on day one (white columns) and day five (grey columns) determined by viability measurements using the Alamar Blue^®^ assay. Columns with error bars represent means + standard deviation (*n* = 4). Means with different letters are statistically significantly different (*p* < 0.05). (**b**,**c**) Corresponding laser scanning microscopy images on day five of cGEL10 (**b**) and cGEL(D)10 (**c**) hydrogels. Fluorescence staining performed: (i) DAPI (blue), stacked image and view of hydrogel frontal plane illustrating nuclei distribution in the reconstructed gel volume; (ii) Alexa Fluor^®^ (Thermo Fisher Scientific, Waltham, MA, USA) 488 phalloidin (green); (iii) merged channels. LSM images represent z-stacks of at least 10 micrographs, and scale bars represent 200 µm.

In this section, various pristine and functionalized dual-component hydrogels were seeded with different cell types. As mentioned earlier, the diversity of ECM within different tissues requires biomaterial adjustments depending on the desired application [27,28]. Thereby, the presence of accessible RGD-like sequences as focal adhesion sites is crucial for integrin-mediated cell adhesion. Additionally, the material stiffness induces a signaling cascade from the outside to the cytoskeleton. Thus, cells sense their microenvironment and respond to adhesion ligands and hydrogel stiffness (mechanosensing). This leads to modulation of cell adhesion, cytoskeleton (re-)organization, and cell phenotype as well as cell viability [11,12]. In a first attempt, L929 mouse fibroblasts, a cell line typically used for initial cytocompatibility assessment, were seeded onto cGEL10 and cGEL(D)10 matrices (Figure 3).

**Figure 4:**
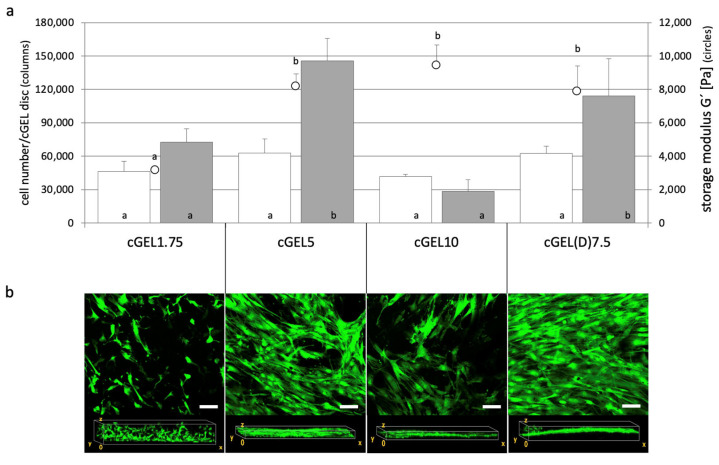
Direct cell contact of human adipose tissue-derived stem cells (hASCs) on cGEL matrices. (**a**) Number of hASCs on hydrogel discs derived from G160 cross-linked with oPNMA-1.75 and COL cross-linked with oPNMA-5, oPNMA-10 and oPDMA-7.5 on day three (white columns) and ten (grey columns), cell numbers were determined by viability measurements (Alamar Blue^®^); storage moduli G’ of cGEL discs rehydrated for 24 h (in PBS, at room temperature) (empty circles). Columns and circles with error bars represent means + standard deviation (columns: *n* = 4; circles: *n* = 5). Means with different letters are statistically significantly different (*p* < 0.05). (**b**) Laser scanning microscopy images (z-stacks of at least 20 micrographs) with Calcein-AM staining on day ten and corresponding view of hydrogel frontal plane. Scale bars in z-stacks represent 100 µm.

Fibroblasts were chosen as they synthesize, arrange, and maintain connective tissue during development or upon (nerve) injury. They offer a vast capacity to differentiate into other cells of the connective tissue, reprogram into nervous tissue (e.g., neurons, SCs), and assist SC migration and neurite regeneration [29,30,31]. Therefore, L929 fibroblast growth behavior was used as an indicator for suitable application in PNR regarding a growth-permissive nature and cytocompatibility of the hydrogels, specifically cGEL10 and cGEL(D)10, in comparison to control conditions on polystyrene tissue culture plate (TCP). The cell growth on TCP was characterized by an increase of one order of magnitude within 24 h. Such a strong initial proliferation was not seen for the cells on the hydrogels. However, on day five, the cell number on cGEL10 and cGEL(D)10 exceeded that found on TCP. Apparently, L929 fibroblast growth and viability came to arrest on TCP due to space constraints. This confirmed the relevance of tissue-related dimensionality of the hydrogel culture system which differed decisively from the monolayer surfaces on TCP [32]. Laser scanning microscopy (LSM) of the hydrogels showed that L929 cells colonized the matrices in multiple planes and showed broad distribution in vertical and horizontal planes. Hence, cell arrangement in a more natural context was supported which characterized the dual-component hydrogels as cytocompatible and growth permissive three-dimensional systems. Among the two hydrogel representatives, higher cell numbers were detected on cGEL10(D) which has two probable causes. First, cGEL10(D) matrices were significantly softer (Table 1) than cGEL10-based matrices and better suited for fibroblasts [11,12]. Second, the methyl ketone functionality in cGEL(D)10 has been shown to transiently immobilize amine-group-containing molecules, and immobilization of serum proteins from the cell culture medium can be assumed [19]. In the following, hASCs were evaluated in direct contact with pristine oPNMA and oPDMA-derived hydrogels to screen a variety of compositions and hydrogel moduli. The interaction with more substrate-selective mesenchymal stem cells was initially screened over a wider range of compositions. To this end, hASCs were seeded on four different gel formulations and cultivated for up to ten days (Figure 4). Cell growth was favorable on all formulations with the highest growth rates on cGEL5 and cGEL(D)7.5. Cell numbers increased by more than one order of magnitude upon day ten; whereby lower proliferation was seen for cGEL1.75 and cGEL10. For COL-based matrices cGEL5, cGEL10, and cGEL(D)7.5, the differences in cell proliferation were irrespective of the determined storage moduli, CLD, and water content. Thus, differences in cell proliferation in this experiment mainly depended on the microstructure of the hydrogel network composed of oligomeric cross-linkers with different anhydride contents, monomer composition (NiPAAm versus DAAm), and gelation conditions (TEA versus NMPO). The reduced cell proliferation on cGEL10 potentially originated from an accumulation of negative net charge that was caused by oPNMA-10 with a high anhydride content. It is commonly known that highly negatively charged surfaces are not favorable for cell-matrix adhesion structures [33].

At this point, G160-based hydrogel formulations were considered less suitable for the ongoing material development for PNR, and the hydrolyzed peptide structure COL was preferred instead. It was not possible to increase the G160 peptide solution concentration above 7.5% without causing physical gelation, which limits the desired tunability of the materials. In contrast, COL’s concentration could be raised to 30% without gelation, allowing for a wider range of hydrogel formulation with the oligomeric cross-linker. The higher concentration of COL provided more amine functions for gelation and cell binding. Additionally, a double syringe system was previously identified for dosing hydrogels, but due to issues of fast gelation and miscibility limitations with TEA, NMPO was selected as a more compatible gelation base, making COL the preferred choice for further formulations [21].

### 2.3. Cellular Response of hASCs with Amine Functionalized cGEL and cGEL(D) Matrices

**Figure 5:**
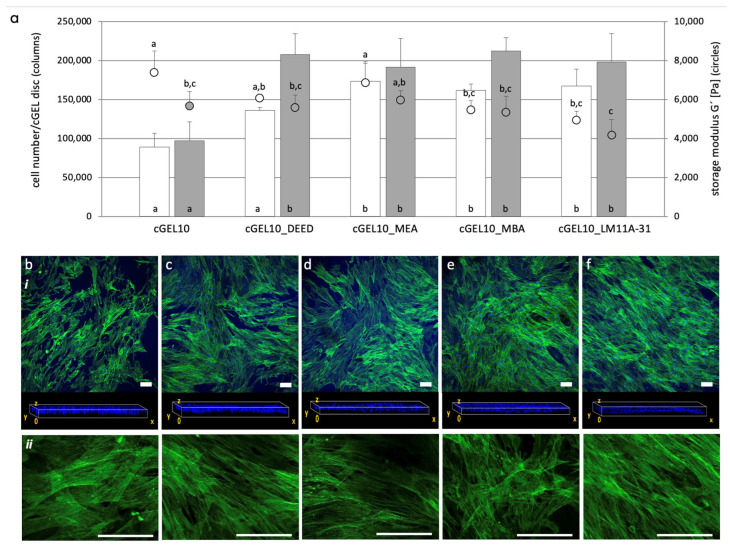
Direct cell contact of hASCs on oPNMA-10-derived cGEL10 and cGEL10_amine. (**a**) Number of hASCs on hydrogels on day three (white columns) and day seven (grey columns), cell number quantified by Alamar Blue^®^ cell viability assay. Storage moduli G’ of rehydrated cGEL discs (in cell culture medium at 37 °C) after day three (empty circles) and day seven (grey circle). Derivatization of cGEL10_amine with DEED: *N,N*-diethylethylendiamine, MEA: 4-(2-aminoethyl)morpholine, MBA: 4-(morpholin-4-yl)butan-1-amine, and LM11A-31: (2S,3S)-2-amino-3-methyl-*N*-[2-(4-morpholinyl)ethyl] pentanamide. Columns and circles with error bars represent means + standard deviation (columns: *n* = 4; circles: *n* = 5). Means with different letters are statistically significantly different (*p* < 0.05). (**b**–**f**) Laser scanning microscopy images (z-stacks of at least 20 micrographs) of (**b**) cGEL10, (**c**) cGEL10_DEED, (**d**) cGEL10_MEA, (**e**) cGEL10_MBA, and (**f**) cGEL10_LM11A-31, (i) cell staining on day seven with Alexa Fluor^®^ 488 Phalloidin (green) and DAPI (blue); corresponding view of cell nuclei distribution in hydrogel frontal plane is shown. Representative single stacks with F-actin fluorescence (ii) are depicted to visualize cell morphology. Scale bars in z-stacks and single stacks represent 100 µm.

To design biomaterials that mimic native cellular environments, we integrated small molecular amines into cGEL10, a hydrogel matrix with low biological performance. This aimed to improve cellular behavior by amine derivatization. The high anhydride content of oPNMA-10 allowed stable cross-linking and mechanical properties for amine integration. Various small molecular amines were covalently incorporated into cGEL10 and assessed for hASC adhesion, proliferation, and migration. By day three, cell adhesion and initial proliferation significantly improved on all cGEL10_amine matrices compared to pristine cGEL10 (Figure 5).

**Figure 6:**
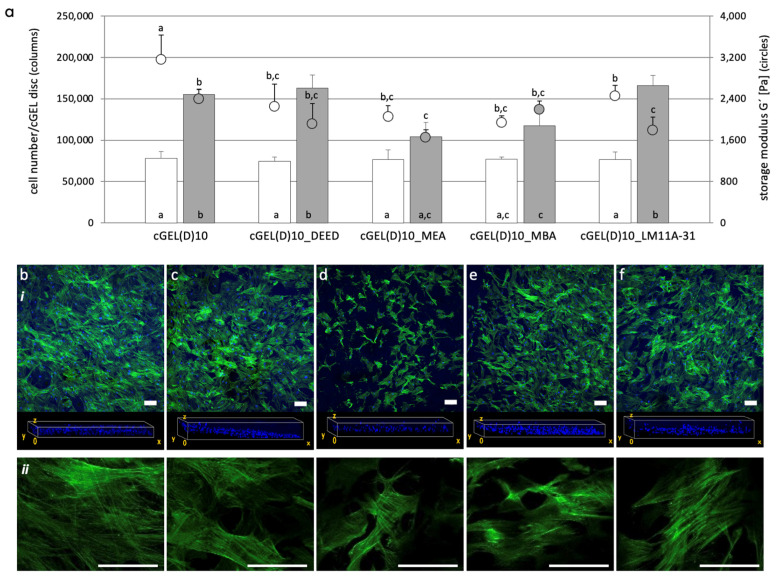
Fate of hASCs on oPDMA-10 derived cGEL(D)10 and cGEL(D)10_amine. (**a**) Number of hASCs on hydrogels on day three (white columns) and day seven (grey columns), cell number quantified by Alamar Blue^®^ cell viability assay. Storage moduli G’ of rehydrated cGEL discs (in cell culture medium at 37 °C) after day three (empty circles) and day seven (grey circle). Derivatization of cGEL(D)10_amine with DEED: *N,N*-diethylethylendiamine, MEA: 4-(2-aminoethyl)morpholine, MBA: 4-(morpholin-4-yl)butan-1-amine, and LM11A-31: (2S,3S)-2-amino-3-methyl-*N*-[2-(4-morpholinyl)ethyl] pentanamide. Columns and circles with error bars represent means + standard deviation (columns: *n* = 4; circles: *n* = 5). Means with different letters are statistically significantly different (*p* < 0.05). (**b**–**f**) Laser scanning microscopy images (z-stacks of at least 20 micrographs) of cGEL(D)10 (**b**), cGEL(D)10_DEED (**c**), cGEL(D)10_MEA (**d**), cGEL(D)10_MBA (**e**), cGEL(D)10_LM11A-31 (**f**), (i) cell staining on day seven with Alexa Fluor^®^ 488 phalloidin (green) and DAPI (blue); corresponding view of cell nuclei distribution in hydrogel frontal plane. Representative single stacks with F-actin fluorescence (ii) are depicted to visualize cell morphology. Scale bars in z-stacks and single stacks represent 100 µm.

This observation cannot only be attributed to softer cGEL10_amine hydrogels as the corresponding storage moduli at day three did not significantly differ from cGEL10 in all cases. The cell numbers further increased until day seven; whereby equivalent cell numbers were measured on all functionalized composites irrespective of the coupled amine and storage modulus. However, on pristine cGEL, the cell proliferation came to arrest and cell number stalled between days three and seven. Regarding cell migration into the hydrogel, the frontal plane view on day seven visualized the distribution of cell nuclei throughout the hydrogel. For cGEL10 and cGEL10_amine, similar cell migration and distribution in the hydrogel bulk were seen. Regarding cell elongation, the alignment along the matrix was supported in cGEL10_amine and profound for cGEL10_LM11A-31. This observation was in accordance with guided cell growth that was seen before upon LM11A-31 integration [16]. It can be hypothesized that cell growth was affected by the integrated amines, thus cGEL10_amine had a biological effect on the seeded cells. Most likely, these amines provided focal adhesion sites for initial adhesion to the surface and contact points for accelerated spreading, alignment, and migration. All grafted amines imparted non-nucleophilic tertiary amino groups that were accessible for protonation and partial neutralization of anionic groups under culture conditions. As cell adhesion-mediating molecules, such as fibronectin, provide anionic charges, their adsorption to positive/neutral surface structures and subsequent attachment of cell adhesion receptors was likely mediated [33]. Additionally, cell adsorption was supported by non-specific binding between cationic moieties on the hydrogel and negatively charged glycocalyx on the cell surface [8,34]. As discussed earlier, the amine functionalization increased the water content of the hybrid hydrogels which also influenced the cellular behavior within the hydrogel. Considering the storage moduli for cGEL10 and cGEL10_amine, the decreased matrix stiffness upon amine integration after days three and seven was mentioned earlier. There was no dependence between the storage moduli and the number of detected cells for both time points. Thus, the biochemical effect of the grafted amines was paramount for the hydrogel properties. It was further visible that the hybrid hydrogels did not significantly degrade under cell culture conditions within the monitored time range. However, as the storage moduli were measured on the cell-free hydrogels, any dynamic cell-matrix reciprocity with the cell-mediated remodeling of the matrices was not visible at this point [8,28]. Regardless of this limitation, these findings were in consensus with published data on cGEL10_DEED and the aligned growth of human sweat gland-derived stem cells in contrast to cGEL7.5 and cGEL10 [21]. The experimental set-up with hASCs was repeated for oPDMA-derived hydrogels. At first, oPDMA-7.5 was chosen based on the promising initial cell proliferation (Figure 4) and the potential acceleration of the cell response by amine integration (in Supplementary Materials Figure S2). In brief, cell proliferation was enhanced on cGEL(D)7.5_DEED and cGEL(D)7.5_LM11A-31. Storage moduli for all cGEL(D)7.5_amine hydrogels were significantly lower than those of oPNMA-7.5 and oPNMA-10 (Figure 5) derived composites. In the following step, oPDMA-10 was pre-functionalized with DEED, MEA, MBA, and LM11A-31, and hASCs were seeded onto the corresponding matrices. The data on cell adhesion, proliferation, and migration on day three and day seven are depicted in Figure 6.

After day three, the number of cells increased by a factor of eight for all hydrogel composites, but the absolute cell numbers were considerably lower than seen on cGEL10 and cGEL10_amine on day three (Figure 5). In addition, there was no difference in cell growth between pristine and functionalized cGEL(D)10. At day seven, the number of hASCs increased tremendously on cGEL(D)10, cGEL(D)10_DEED, and cGEL(D)10_LM11A-3 though the absolute cell numbers did not yield or exceed cell numbers seen for cGEL10_amine analogs. Possible explanations for the enhanced cell proliferation on pristine cGEL(D)10 have been discussed earlier. The cross view of the frontal plane indicated the distribution of hASC nuclei throughout the different hydrogels. More cells migrated into the gels in cGEL(D)10_DEED, cGEL(D)10_MBA, and cGEL(D)10_LM11A-31 samples. In comparison, hASCs were more loosely dispersed in cGEL10(D) and cGEL(D)10_MEA. On the material level, the storage moduli of oPDMA-10-based gels were significantly lower than corresponding oPNMA-10 derivatives (refer to Figure 5a and Figure 6a) for both time points. All investigated oPNMA-10- and oPDMA-10-derived hydrogels exhibited reduced storage moduli upon amine integration and no direct correlation to cell growth was discernable. Thus, the diverse cell proliferation profiles are attributed to the hydrogel structure with the different oligomer cross-linkers as well as the integrated amine molecules rather than the hydrogel stiffness. A graphical summary of the overall three investigated hydrogel systems with a focus on cell proliferation and hydrogel stiffness is given in Supplementary Materials Figure S4. In general, cell proliferation and migration were profound on cGEL10_amine and reproducible for DEED and LM11A-31 derivatives of cGEL and cGEL(D). Cell growth and viability were overall mainly affected by the type of integrated amine and no direct correlation between cell proliferation and material stiffness was found. In consensus with former data, however, the higher stiffness of all cGEL10 composites potentially contributed to the observed increased cell spreading [9]. In addition to the direct cell-matrix interaction, indirect cytocompatibility tests were performed on the hydrogel extracts of pristine and modified cGEL10, cGEL(D)10, and cGEL(D)7.5 according to published protocols [16,18]. All extracts showed high cell metabolic activities above 70% relative to the positive live control on TCP after 24 h and 72 h (refer to Supplementary Materials Figure S3). Thus, there was no evidence of toxic leachability as a possible result of inefficient oligomer conversion during cross-linking or rapid release of early incompatible degradation products. In accordance with the high CLD for all formulations, this finding reaffirmed an efficient integration of the hydrogel components. The regenerative potential of the presented hybrid hydrogel systems in combination with hASCs is based on the multi-lineage differentiation capability of ASCs that might be directed by adjusted biophysical, biochemical, or biological properties of the hydrogels [9,35]. With a specific focus on PNR, hASCs have been (i) differentiated into neuronal-like cells (ii) assembled to form neurospheres, (iii) self-aligned and co-cultured with neurons, (iv) loaded into enriched nerve guidance conduits, and (v) formed into scaffoldless tubes after differentiation [36,37,38,39,40]. This motivates the prospective application of hASCs on cGEL composites with functionalized small molecular amines, signal molecules, or morphogenes to enhance PNR.

### 2.4. Cellular Response and Interaction of Neonatal Schwann Cells with cGEL Hydrogels

**Figure 7:**
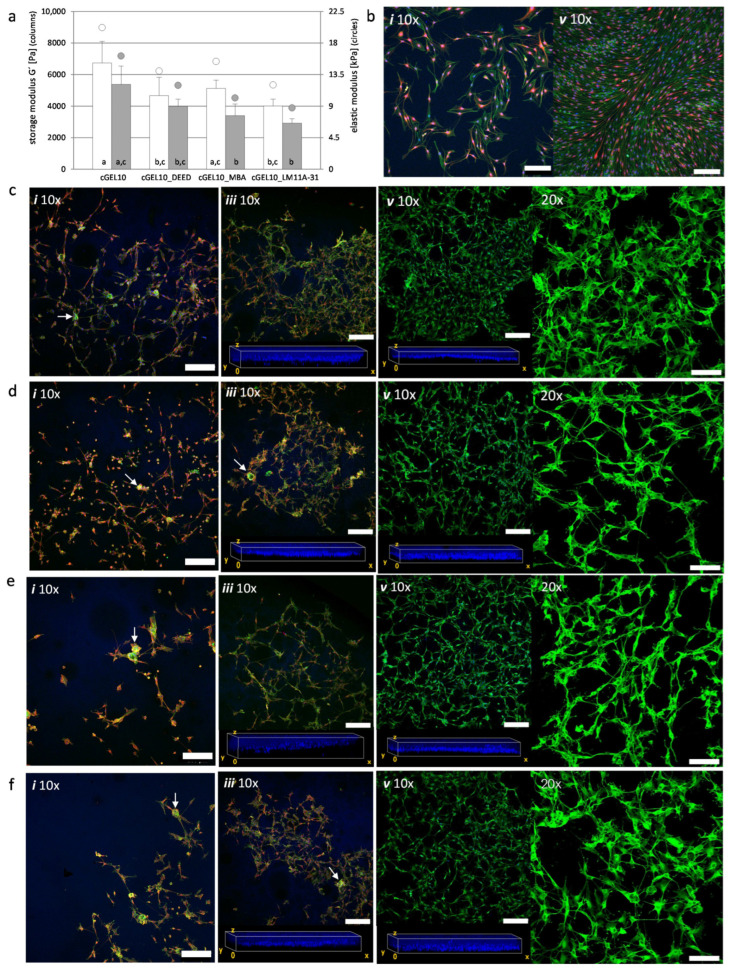
Direct cell contact of neonatal rat Schwann cells (nSCs) on oPNMA-10 derived cGEL10 and cGEL10_amine hydrogels. (**a**) Storage moduli G’ (columns) and derived elastic moduli E (circles) of rehydrated cGEL10 and cGEL10_amine (DEED, MBA, and LM11A-31) discs after day one (white) and day five (grey) (rehydrated in cell culture medium, 37 °C). Columns with error bars represent means + standard deviation (*n* = 5). Columns with different letters are statistically significantly different (*p* < 0.05). (**b**–**f**) NSCs seeded on PLL-coated tissue culture plate (**b**), cGEL10 (**c**), cGEL10_DEED (**d**), cGEL10_MBA (**e**), and cGEL10_LM11A-31 (**f**). LSM images on day one (i), three (iii), and five (v) with tenfold magnification (10×, z-stacks of at least 15 micrographs): fluorescence staining is shown with Alexa Fluor^®^ 488 phalloidin (green), DAPI (blue), and alpha-S100/Alexa Fluor^®^ 546 (red, for hydrogels only on day one and three); cluster formation (white arrows); (iii,v): corresponding view of cell nuclei distribution in hydrogel frontal plane; twentyfold magnification (20×, z-stacks of at least 10 micrographs): fluorescence staining is shown with Alexa Fluor^®^ 488 phalloidin (green). Scale bars in z-stacks represent 200 µm (10×) and 100 µm (20×).

Schwann cells (SCs) are derived from neural crests and form principal glial cells in the peripheral nervous system. As myelinating or non-myelinating cells they support, modulate, and maintain axonal function and survival [41]. During PNR, the expression of neurotrophic factors and ECM proteins by so-called repair SCs is increased and allows sensing of the microenvironment [42,43,44]. Thereby, peripheral nerve cells are modulated with regard to motility and differentiation in response to the physical environment and specifically material stiffness [27,45,46]. For this experiment, the storage moduli of cGEL10, cGEL10_amine (DEED, MBA, LM11A-31) were measured on days one and five, the elastic modulus (E) was estimated from storage moduli obtained (E = 3G’) and compared to published data (Figure 7a) [47]. The initial modulus of pre-functionalized cGEL10_amine (E < 15.5 kPa) was lower than that of pristine cGEL10 (20 kPa) and decreased for all formulations upon day five by 15% to 35%. In general, the hydrogel material should be comparable to connective tissue that surrounds peripheral nerves. On the other hand, SC response is known to depend on the chemical composition and microstructure of the substrate [48,49]. As SCs show high mechanosensitivity over a broad range of moduli covering hundreds Pa to MPa in dependence on the system’s dimensionality, the herein presented gelatin-derived hydrogels offer a suitable material platform as substrates for SCs [50,51,52]. In the following, nSCs were seeded on PLL-coated TCP, cGEL10, and cGEL10_amine composites. Based on the inconsistent literature data, a density of 150 cells/mm^2^ was selected as an intermediate value of sub-confluent cells (reported cell densities: 10–10^3^ cells/mm^2^) to allow for cell adhesion, viability, and proliferation [53,54,55,56]. In control wells, the polymorphic appearance of nSCs changed into highly aligned swirls with flattened cell bodies and long extensions while cell purity was conserved (positive alpha-S100) (Figure 7b). This cell monolayer likely originated from cell–cell contact inhibition with senescence avoidance and morphological response. In consensus with other reports, the directional swarm formation of nSCs on PLL-coated TCP was not replicated by SCs on and within all tested hydrogels [45,50,51,55].

In this context, direct comparison of cell morphology and proliferation rate between 2D TCP and 3D systems is limited due to different availability of nutrients, gases, effector molecules, and variable replication of nervous tissue [44,55,57]. At day one, the cell morphology within the hydrogels ranged from elongated with short protrusions to mostly spherical with cluster formation. The cell elongation was especially more pronounced on cGEL10 as compared to softer cGEL10_amine hydrogels (Figure 7c–f). The cell clusters disappeared in all hydrogel specimens as SCs migrated out of the bundles and started to show different morphologies from polarized, spindle-like to spread with multiple protrusions (unpolarized). In all composites, nSCs developed extensions that either spawned fingerlike protrusions into the matrix or formed direct connections to surrounding cells in all orientations. The strong fluorescent signal for F-actin (Figure 7c–f, 20× magnification) indicated a vertical and horizontal cell-cell interaction and the interplay between external culturing conditions that resulted in stress fiber formation [12,45,50]. In all hydrogel compositions, lamellar protrusions with contact tips were exposed. This could either indicate focal adhesion or growth constraints though neither of these phenomena was yet postulated in comparable 3D culture to the best of our knowledge. In addition, the migration tendency was enhanced within cGEL10_amine hydrogels which is in accordance with observations for increased hASC distribution. Taken together, cGEL10 and cGEL10_amine hydrogels allowed adherence, migration, proliferation, and viability of nSCs in the neuron-free culture. Thus, promising hydrogel matrices were herein presented. At this point, it was not possible to distinguish the effect of amine functionalization on the cell behavior; though the overall performance was promising compared to the literature data on enriched hydrogels with Matrigel^TM^ (Corning Inc., Corning, NY, USA)or growth factors, as well as reports on hydrogel systems embedded with SCs based on poly(ethylene glycol), fibrin or hyaluronic acid-laminin [51,55,58,59]. The (partial) neutralization of the anionic charge surplus in cGEL10 by amine integration resulted in various cell morphologies with a substantial increase of elongated and spindle-like cell bodies in cGEL10_DEED and cGEL10_MBA. The growth impact of LM11A-31 (NGF mimetic) was not clearly visible in the primary culture. Thus, the incorporation of LM11A-31 in cGEL did not lead to the chemoattraction of SCs which was also not reported for NGF, though any chemokinetic or myelinating effects were not investigated at this point [60,61]. This is of importance and needs to be addressed in further investigations since SCs shift between a myelinating and non-myelinating phenotype which has an effect on the material-cell-driven interactions and the myelination abilities in the context of PNR [41]. Regarding the chemical integrity of LM11A-31 upon grafting to oPNMA, it likely affected the binding sites to the important neurotrophic receptor p75 on nSCs. In detail, the primary amine in LM11A-31 was consumed by amide formation to oPNMA and, thus, was unavailable as a hydrogen bond donor or positive ionizable group for receptor p75 activation. But other hydrogen bond donor (amide bond) and acceptor (morpholine oxygen) groups were conserved, and additional binding sites, such as amides derived from NiPAAm in the oligomeric cross-linkers or ammonium groups as the protonated tertiary amine of morpholine, were introduced [62]. The same was true for the morpholine derivate MBA in cGEL10_MBA which showed enhanced biological performance due to the introduction of the morpholine structure. The overall biochemical impact of the investigated amines might be pronounced in multicell-type/co-culture systems or in nerve lesion models. In extension to the presented amines, the covalent incorporations of bioactive molecules such as cyclic adenosine monophosphate, amino acid motif of laminin-2, glycoproteins (e.g., fibronectin), mitogenic stimuli (e.g., neuregulin-1), or basal lamina proteins (e.g., laminin) offer huge potential in cGEL hydrogel design for improved remyelination [58,63,64,65]. Furthermore, the integration of topographical cues such as nanofibers or longitudinal capillaries is strongly suggested for the optimization of cGEL systems for enhanced PNR [64,65,66].

## 3. Materials and Methods

### 3.1. Materials

Pentaerythritol diacrylate monostearate (PEDAS), N-isopropylacrylamide (NiPAAm), diacetone acrylamide (DAAm), 2,2-azobis(2-methylpropionitrile) (azobisisobutyro-nitrile, AIBN), N-methylpiperidin-3-ol (NMPO), (2S,3S)-2-amino-3-methyl-N-[2-(4-morpholinyl)ethyl]pentanamide dihydrochloride (LM11A-31*2HCl), *N,N*-diethylethylendiamine (DEED), 4-(*N,N*-diethylamino)-butylamine (DEAB), 4-morpholinoethylamine (MEA), and 2,4,6-trinitrobenzenesulfonic acid (TNBS) were purchased from Sigma-Aldrich (Seelze, Germany). 4-Morpholinobutylamine (MBA) was purchased from Santa Cruz Biotechnology (Heidelberg, Germany). Maleic anhydride (MA), triethylamine (TEA) and sodium hydroxide were obtained from Acros Organics (Geel, Belgium). Fractions of partially hydrolyzed gelatinous protein, Collagel^®^ (type B, 11.5 kDa, viscosity (10% (*w*/*v*), 25 °C): 3.58 mPas, Lot # 895046, COL) and Gelatin type B bloom 160 (G160) (batch #630253MI00) were kindly provided by Gelita AG (Eberbach, Germany). *N,N*-Dimethylformamide (DMF), and phosphate-buffered saline (PBS, composition KCl: 0.2 g/L, KH_2_PO_4_: 0.2 g/l, NaCl: 0.8 g/l, Na_2_HPO_4_ anhydrous: 1.15 g/l; pH 7.4) were purchased from AppliChem (Darmstadt, Germany). Dulbecco’s modified eagle medium with low glucose (DMEM lg) or high glucose (DMEM hg) with or without phenol red, alanyl-glutamine solution (200 mM), TritonTM X-100, bovine serum albumin (BSA), goat serum (GS), and poly-L-lysine hydrobromide (PLL, molecular weight > 300,000) was purchased from Sigma Aldrich (Seelze, Germany). Fetal bovine serum (FBS), penicillin/streptomycin (P/S), and trypsin were purchased from PAA Laboratories (Pasching, Austria). For extract filtration, Rotilabo^®^ polyethersulfone (PES) filters (0.2 µm pore size) were purchased from Carl Roth^®^ (Karlsruhe, Germany). Precision silicon O-rings were bought from RS Components GmbH (Mörfelden-Walldorf, Germany) and HUG^®^ Technik und Sicherheit GmbH (Ergolding, Germany). Alamar Blue^®^ reagent, Calcein-acetoxymethyl (Calcein-AM), 4’,6-diamidino-2-phenylindole/dihydrochloride (DAPI), Alexa Fluor^®^ 488 phalloidin, and the secondary antibody Alexa Fluor^®^ 546 anti-rabbit IgG (H + L) were purchased from Invitrogen (Darmstadt, Germany). The primary antibody polyclonal rabbit (alpha-S100, Dako Omnis) was purchased from Agilent (Waldbronn, Germany) and 7-deacetyl-7-(o-(N-methylpiperazino)-gamma-butyryl)-dihydrochloride (Forskolin) from Merck KGaA (Darmstadt, Germany).

### 3.2. Oligomeric Cross-Linker Synthesis and Characterization

Oligomeric cross-linkers, oligo(PEDAS-co-NiPAAm-co-MA) (oPNMA) and oligo(PEDAS-co-DAAm-co-MA) (oPDMA), with different anhydride contents, were synthesized as published [18,19]. Briefly, comonomer mixtures of PEDAS, NiPAAm, or DAAm and MA were copolymerized by free radical polymerization with AIBN in THF (60 °C, nitrogen atmosphere). In detail, PEDAS was copolymerized with MA and NiPAAm or DAAm in a constant molar ratio of 1:20. The resulting oligomeric macromers were abbreviated as oPNMA-x and oPDMA-x with x representing the molar ratio of MA in the comonomer feed relative to PEDAS. After synthesis, oligomers were precipitated in diethyl ether and vacuum dried. The chemical compositions, molecular weight distributions, and fraction of chemically intact anhydride groups were determined by titration as published [18,19]. 

### 3.3. Functionalization of oPNMA-x and oPDMA-x with Small Molecular Amines

The pre-derivatization of oPNMA-x in a simple procedure has been described [15,21]. Shortly, oligomers oPNMA-10, oPDMA-7.5, and oPDMA-10 were functionalized in an amidation reaction with a defined ratio of primary amine-functionalities of five different small molecular amines. The derivatization degree was selected to find a compromise between sufficient derivatization to achieve a biological effect and maintain enough residual anhydride groups for effective cross-linking of Collagel^®^ (COL). In the designed set-up, normalized equivalents of chemically intact anhydride groups (MAeq) in oPNMA-x and oPDMA-x were coupled with *N,N*-diethylethylendiamine DEED), 4-(*N,N*-diethylamino)-butylamine (DEAB), 4-(2-aminoethyl)morpholine MEA), 4-(morpholin-4-yl)butan-1-amine (MBA), (2S,3S)-2-amino-3-methyl-N-[2-(4-morpholinyl)ethyl]pentanamide (LM11A-31) to yield the derivatized oligomers oPNMA-x + amine and oPDMA-x^+amine^ (e.g., oPNMA-10^+DEED^). During derivatization, the amine was added in a molar ratio expressed as anhydride equivalents (MAeq, *n*) that corresponds to *n* times the normalized molarity of anhydrides in the oligomer solution calculated from the content of chemically intact anhydrides per oligomer divided by the molar mass of maleic anhydride. Thereby, MAeq *n* expresses that the amine is added to a percentage that equals *n* moles per chemically intact anhydride in the oligomer, e.g., 10 in oPNMA-10 or 7.5 in oPDMA-7.5 [16]. For DEED, DEAB, MEA, and MBA, the derivatization of the oligomer was directly performed from appropriate amine dilutions at ambient conditions for 15 min. For LM11A-31, the dihydrochloride salt was converted into the free base prior to the coupling reaction by the addition of molar equivalent NMPO to neutralize the protonation of the hydrochloride.

### 3.4. Fabrication of Cross-Linked Hydrogel cGEL and cGEL(D)

Cross-linked hydrogels based on gelatin (G160) or Collagel^®^ (COL) were fabricated as published before [15,21]. In brief, aqueous COL (30% *w*/*v*) or G160 (7.5% *w*/*v*) solution was vortexed with oligomeric solution (7% *w*/*v* in DMF) of oPNMA-x or oPDMA-x in the presence of the base TEA (undiluted) or NMPO (20% (*v*/*v*) in water) in volume ratio of 4:4:1 of peptide to oligomer to base. After the gelation, hydrogels were dried, washed in (pH = 7.4), and punched into normalized discs [21]. Afterward, hydrogels were lyophilized and sterilized by g-irradiation (13.5–16.5 kGy) at Synergy Health Radeberg GmbH (Radeberg, Germany) or alternatively at BBF Sterilisationsservice GmbH (Kernen, Germany), and rehydrated for 24 h in PBS (pH = 7.4), or cell culture medium. These cross-linked hydrogels are abbreviated as cGEL. Furthermore, they are termed cGELx for oPNMA-x derived and cGEL(D)x for oPDMA-x derived matrices with x depicting the theoretical molar MA content of the utilized cross-linker. For functionalized hydrogels, oPNMA-10, oPDMA-7.5, and oPDMA-10 were derivatized with small amines (MAeq 2.5) prior to the gelation step as described above. The manufactured cGEL discs are abbreviated as cGEL10_amine (based on oPNMA-10), cGEL(D)7.5_amine (based on oPDMA-7.5) and cGEL(D)10_amine (based on oPDMA-10); whereas the extension “amine” represents the integrated amine DEED, MEA, MBA, or LM11A-31. A summary of all cGEL and cGEL(D) compositions used in this study is given in Table 2.

### 3.5. Mechanical Characterization

CGEL and cGEL(D) discs were mechanically characterized according to previous protocols by oscillation rheology with a thermostatic rheometer (Physica MCR 301, Anton Paar, Graz, Austria, storage and loss moduli were recorded by the RHEOPLUS software (Anton Paar, Version 3.4) [21]. In brief, the rheometer was equipped with an 8 mm steel plate and rehydrated discs (∅ 8 mm, reconstituted for 24 h in PBS at ambient conditions) were measured under normal force (0.2 N) and temperature control (37 °C). A frequency sweep test was performed on the hydrogels from 0.1 to 10 Hz. The storage modulus (G’) was depicted at a frequency of 1 Hz (average of five discs, *n* = 5). This measurement was also performed for cGEL and cGEL(D) discs rehydrated in cell culture medium for one to seven days in a humidified incubator at 37 °C.

### 3.6. Cross-Linking Degree and Water Content

Cross-linking degree: Residual amine functionalities in cGEL and cGEL(D) were determined according to a published assay using 2,4,6-trinitrobenzenesulfonic acid (TNBS) [15,21]. Thereby, lyophilized hydrogel fractions were immersed in a mixture of 4 parts water, 1 part of 4% (*w/v*) aqueous sodium bicarbonate (pH 8.5), and one part 0.5% (*v/v*) aqueous TNBS solution. Afterward, sample solutions were heated (4 h at 40 °C) and hydrolyzed in 6 N HCl solution (1.5 h at 60 °C) and the absorbance of the solutions was measured spectrophotometrically at 340 nm (Spectronic Genesys 6, Thermo Electron Scientific Instruments LLC, Madison, WI, USA). For reference, powdered COL (or G160 in the case of cGEL1.75) was processed and quantified accordingly. For blank values, the formation of the chromogenic amine was avoided by HCl addition before TNBS was added. The cross-linking degree (CLD) for COL-based hydrogels was calculated by Equation (1) [15]. All measurements were performed in triplicate.

(1) $$\mathrm{CLD}\;(\%)\;=\left(1-\left(\frac{\mathrm{absorbance}\;\mathrm{cGEL}}{\mathrm{absorbance}\;\mathrm{COL}}\right)\right)\times 100$$

Water content: Swollen hydrogel discs (∅: 8–12 mm) were weighted (wet weight, WW), frozen at −20 °C, and lyophilized [15]. Based on the resulting dry weight (DW) the water content was calculated according to Equation (2). All measurements were performed in quintuplicate.

(2) $$\mathrm{water}\;\mathrm{content}\;(w/w)\;=\frac{\left(\mathrm{WW}-\mathrm{DW}\right)}{\mathrm{DW}}$$

### 3.7. In Vitro Cell Proliferation of Fibroblasts and Adipose Tissue-Derived Stem Cells on Pristine and Amine-Functionalized cGEL and cGEL(D)

The proliferation of L929 mouse fibroblasts (Cell Lines Service, Eppelheim, Germany) and human adipose tissue-derived stem cells (hASC; EK-BR-9/13-1) was analyzed as published [16]. Briefly, L929 (passage 42) and hASCs (passages 5 to 7) were seeded onto the hydrogel formulations: cGEL1.75, cGEL5, cGEL10, cGEL(D)7.5, cGEL(D)10 as well as amine-functionalized cGEL10_amine, cGEL(D)7.5_amine, and cGEL(D)10_amine. Thereby, sterilized hydrogel discs (∅ 11 mm) were reconstituted in DMEM lg and 1% (*v*/*v*) P/S in 48 well plates for 24 h at 37 °C. A silicone O-ring was inserted in each well to keep the swollen discs submerged in media. Afterward, 10^4^ cells per hydrogel and well (0.95 cm^2^) were seeded in cell culture medium (DMEM lg, 10% FBS, 1% P/S) and incubated (37 °C, 5% CO_2_ atmosphere). The culture medium was exchanged every other day. Cell viability was measured via Alamar Blue^®^ (Bio-Rad Laboratories, Hercules, CA, USA) assay for L929 after days one and five and for hASCs after days three and ten (*n* = 4). For microscopic observation, two fluorescence-staining protocols were performed. Firstly, cells were stained with 2 µM Calcein-AM as described elsewhere [16]. Alternatively, cells were washed in PBS, fixated (3.7% paraformaldehyde (*v*/*v*)), permeabilized (0.5% (*v*/*v*) Triton X-100), and stained with DAPI (300 nM, 1:1000) and Alexa Fluor^®^ 488 phalloidin (1:40) according to the manufacturer’s protocol. Hydrogels were microscopically examined with laser scanning microscopy (LSM 700, Zeiss, Germany) and z-stacks were reconstructed from at least 10 (L929) or 20 (hASC) individual micrographs. The cell nuclei (DAPI) or cell body (Calcein-AM) distribution in the matrices is shown by the 3D reconstruction of the gels. In all cases, the range of z-stacks did not exceed the hydrogel height but covered more than 50% of the cross-section so that the core is always included in the reconstructions.

### 3.8. Neuron-Free Culture and Migration of Neonatal Schwann Cells (nSC)

The nSCs were harvested from animals sacrificed for scientific purposes in accordance with §4 of the German animal protection law and with the European Communities Council Directive 2010/63/EU for the protection of animals used for experimental purposes. All harvest was registered with the Local Institutional Animal Care and Research Advisory (Registration code: 11/§4/Haastert-Talini-1 and §4 2017/167) and reported to the animal care committee of Lower-Saxony, Germany [67,68]. For primary cell cultivation, tissue culture flasks were coated with PLL in accordance with the manufacturer’s protocol. NSCs were cultured in an expansion medium (CMexp) consisting of DMEM hg with 2 mM glutamine, 10% FBS (*v*/*v*), 1% P/S (*v*/*v*), and 2 µM Forskolin [23]. The medium was exchanged every other day and cells were split when confluent. For cell migration measurement, nSCs (passage 11) were seeded on pristine and amine-functionalized hydrogel discs cGEL10. Sterilized discs (∅ 11 mm) were rehydrated with DMEM hg and 1% (*v*/*v*) P/S (24 h, 37 °C). Silicone O-rings were used as described above in low-adhesion 48 well plates. Afterward, 104 cells per hydrogel (0.64 cm2) were seeded in CMexp and incubated (37 °C, 5% CO2 atmosphere). For positive controls, the same cell density was seeded in PLL-coated wells. CMexp was exchanged every other day and cell migration was visualized on days one, three (only for cGEL10), and five by fluorescence staining. Therefore, matrices and control wells were washed, and cells were fixed and permeabilized in a blocking solution containing bovine serum albumin (BSA) and goat serum (GS). Furthermore, cells were incubated in diluted blocking solution with the primary antibody polyclonal rabbit alpha-S100 (1:200) and secondary antibody Alexa Fluor^®^ 546 anti-rabbit IgG (H + L) (1:500) [69]. For nucleus counterstaining, nSCs were incubated with DAPI (1:1000) and Alexa Fluor^®^ 488 phalloidin (1:40). For cGEL at day five, cells were only stained with DAPI/Alexa Fluor^®^ 488 phalloidin. Hydrogel discs and control wells were examined with LSM using tenfold (10×) magnification. Z-stacks were reconstructed from at least 15 individual micrographs. Additionally, cell nuclei distribution (DAPI) in the matrices was shown by 3D reconstruction of the hydrogel. Additionally, cells on cGEL matrices were captured with twentyfold magnification (20×) using the phalloidin channel. The resulting z-stacks were reconstructed from at least 10 micrographs.

## 4. Conclusions

The study aimed to enhance the bioactivity of dual-component hydrogels by grafting small heterobifunctional amines. Aliphatic diamines were integrated to neutralize the anionic charge surplus, while NGF-mimetic LM11A-31 and morpholine derivatives were included to enhance PNR. Material characterization showed that the choice of cross-linker, pre-derivatization, and grafted amine influenced properties such as water content and hydrogel moduli. The hydrogel platform was evaluated for cell compatibility, with fibroblasts showing proliferation and migration on certain hydrogels. Hydrogels based on specific cross-linkers were tested for hASC proliferation, revealing that cell performance depended more on grafted molecules than mechanical properties. Neonatal SCs adhered, migrated, proliferated, remained viable, and adapted morphologically on specific functionalized hydrogels. However, a precise response to hydrogel properties has not yet been established. The hybrid hydrogels in this study show promise for biomedical applications, especially in PNR and nerve implant design. Advances in materials and techniques support their use in advanced prototyping and biochemical cue implementation for enhanced PNR.

## Figures and Tables

**Table 1:** Material properties of cGEL and cGEL(D) matrices. Cross-linking degree, water content, and selection of storage moduli determined for different dual-component hydrogels composed of gelatinous peptide and oPNMA-x or oPDMA-x. CLD: cross-linking degree; values of CLD and water content represent means ± standard deviation (*n* = 3). G’ measured after three days in cell culture medium unless indicated; values represent means ± standard deviation (*n* = 5) rounded to decade.

Hydrogel Specimens	CLD [%]	Water Content [*w*/*w*]	Storage Modulus G’ [Pa]
cGEL1.75	88.9 ± 0.7	7.6 ± 0.1	1320 ± 210 *
cGEL5	92.1 ± 1.4	6.3 ± 0.5	8170 ± 760 *
cGEL7.5	90.7 ± 0.9	6.8 ± 0.8	5630 ± 310
cGEL10	92.4 ± 1.0	5.2 ± 0.2	9420 ± 1240 * 7370 ± 1120
cGEL10_DEED	82.0 ± 2.3	7.8 ± 0.3	6050 ± 140
cGEL10_MBA	82.5 ± 1.5	7.7 ± 0.5	5450 ± 480
cGEL10_LM11A-31	80.8 ± 1.3	8.2 ± 0.6	4930 ± 460
cGEL(D)7.5	93.1 ± 0.8	5.8 ± 0.3	7870 ± 1530 * 3350 ± 430
cGEL(D)10	80.4 ± 2.2	10.2 ± 0.8	3160 ± 470
cGEL(D)10_DEED	83.4 ± 2.1	13.7 ± 0.7	2250 ± 440
cGEL(D)10_MBA	74.2 ± 1.5	14.9 ± 1.3	1940 ± 130
cGEL(D)10_LM11A-31	77.3 ± 2.2	11.9 ± 0.4	2460 ± 200

* After 24 h in phosphate-buffered saline.

**Table 2:** Overview of fabricated cross-linked cGEL and their compositions with and without derivatization of small molecular amines. G160: gelatin boom 160; COL: Collagel^®^; TEA: triethylamine, NMPO: *N*-methylpiperidin-3-ol; x: molar ratio of maleic anhydride (MA) to PEDAS in oPNMA-x/oPDMA-x in synthesis feed; DEED: *N,N*-diethylethylendiamine, MEA: 4-morpholinoethylamine, MBA: 4-morpholinobutylamine, LM11A-31: (2S,3S)-2-amino-3-methyl-*N*-[2-(4-morpholinyl)ethyl]pentanamide; cGELx: derived from oPNMA-x, cGEL(D)x: derived from oPDMA-x with x expressing the analog MA content of the cross-linker.

	Peptide		Gelation Base		Oligomeric Cross-Linker				Small Molecular Monovalent Amine			
	G160	COL	TEA	NMPO	oPNMA-x		oPDMA-x		DEED	MEA	MBA	LM11A-31
	[%] ^1^	[%] ^1^	[%] ^1^	[%] ^1^	x	[%] ^2^	x	[%] ^2^	[MAeq] ^3^			
cGEL1.75	3.25		10		1.75	3.5						
cGEL5		15	10		5	5.5						
cGEL7.5		15		2	7.5	3.5						
cGEL10		15		2	10	3.5			2.5	2.5	2.5	2.5
cGEL(D)7.5		15		2			7.5	3.5	2.5	2.5	2.5	2.5
cGEL(D)10		15		2			10	3.5	2.5	2.5	2.5	2.5

^1^ weight percentage during gelation; ^2^ weight percentage in cGEL; ^3^ conversion of *n* out of x equivalents of chemically intact anhydrides in oligomer.

## Data Availability

The data that support the findings of this study are available from the corresponding author upon reasonable request.

## Supplementary Materials

The following supporting information can be downloaded at: https://www.mdpi.com/article/10.3390/ijms26115316/s1.
