# Supplementary material for: Functionalization of Two-Component Gelatinous Peptide/Reactive Oligomer Hydrogels with Small Molecular Amines for Enhanced Cellular Interaction
Source: Int J Mol Sci. 2025 May 31;26(11):5316. doi: 10.3390/ijms26115316 (PMC12155368; doi:10.3390/ijms26115316)
Supplement: Supplementary file 1 [file ijms-26-05316-s001.zip › ijms-3568754-supplementary.pdf]

Supporting Information

Functionalization of two-component gelatinous peptide/reactive oligomer hydrogels with small molecular amines for enhanced cellular interaction

Caroline Kohn-Polster, Benno Müller, Jan Krieghoff, Awais Nawaz, Iram Maqsood-Umar, Annett Starke, Kirsten Haastert-Talini, Michaela Schulz-Siegmund, and Michael C. Hacker**

Oligomeric building block composition [a]					Application in study
		anhydride content		molecular weight	
	#	[*wt*%]	intact [%]	Mn [Da]	utilization in data sets (reference to Figure/Table/SI)
oPNMA-x					
1.75	1	7.5	76.3	4568 ±95	CLD/water content (Table 2); *in vitro* culture hASC on cGEL, rheology (Figure 4)
5	1	15.8	65.9	2873 ±3	CLD/water content (Table 2) *in vitro* culture hASC on cGEL, rheology (Figure 4)
7.5	1	20.0	68.2	2227 ±65	CLD/water content (Table 2); *in vitro* culture hASC on pristine cGEL, rheology, cytocompatibility of cGEL extracts on L929 (SI 2)
10	1	23.8	73.3	1970 ±23	CLD/water content (Table 2); *in vitro* culture hASC on cGEL, rheology (Figure 4)
	2	24.6	66.2	1830 ±12 -- 1986±11*	*in vitro* culture L929 on cGEL (Figure 3); *in vitro* culture hASC on pristine/amine modified cGEL, rheology (Figure 5); cytocompatibility of cGEL extracts on L929 (SI 3)
	3	24.0	78.4	1643 ±15	CLD/water content (Table 2); *in vitro* culture nSC on pristine/amine modified cGEL, rheology (Figure 7)
oPDMA-x					
7.5	1	16.7	72.2	3019 ±26	CLD/water content (Table 2); *in vitro* culture hASC on pristine/amine modified cGEL(D), rheology (SI 2)
10	1	20.2	69.3	2397 ±24	CLD/water content (Table 2); *in vitro* culture L929 on cGEL(D) (Figure 3); *in vitro* culture hASC on pristine/amine modified cGEL(D), rheology (Figure 6); cytocompatibility of cGEL(D) extracts on L929 (SI 3)

[a]Anhydride content, total MA content of synthesized oligomers expressed as weight percent and percentage of chemically intact MA groups; number-average molecular weight (Mn) determined by gel permeation chromatography (GPC), values express means ± standard deviation (n=3), *data not available: range of Mn for two batches before and after synthesis of batch 2; # batch code of synthesized oligomers; CLD: cross-linking degree; hASC: human adipose tissue-derived stem cells; L929: mouse fibroblasts; nSC: neonatal Schwann cells.

Figure S 1 Chemical properties of synthesized and utilized oPNMA-x and oPDMA-x oligomer batches and the application of deviated cGEL and cGEL(D) matrices within this study. The analysis of oPNMA-x and oPDMA-x batches showed that macromer properties of oPNMA-x and oPDMA-x (i.e., anhydride content and molecular weight Mn) were reliable and reproducible irrespective of the anhydride content. Therefore, hydrogel properties were not biased by inhomogeneous macromer batches.

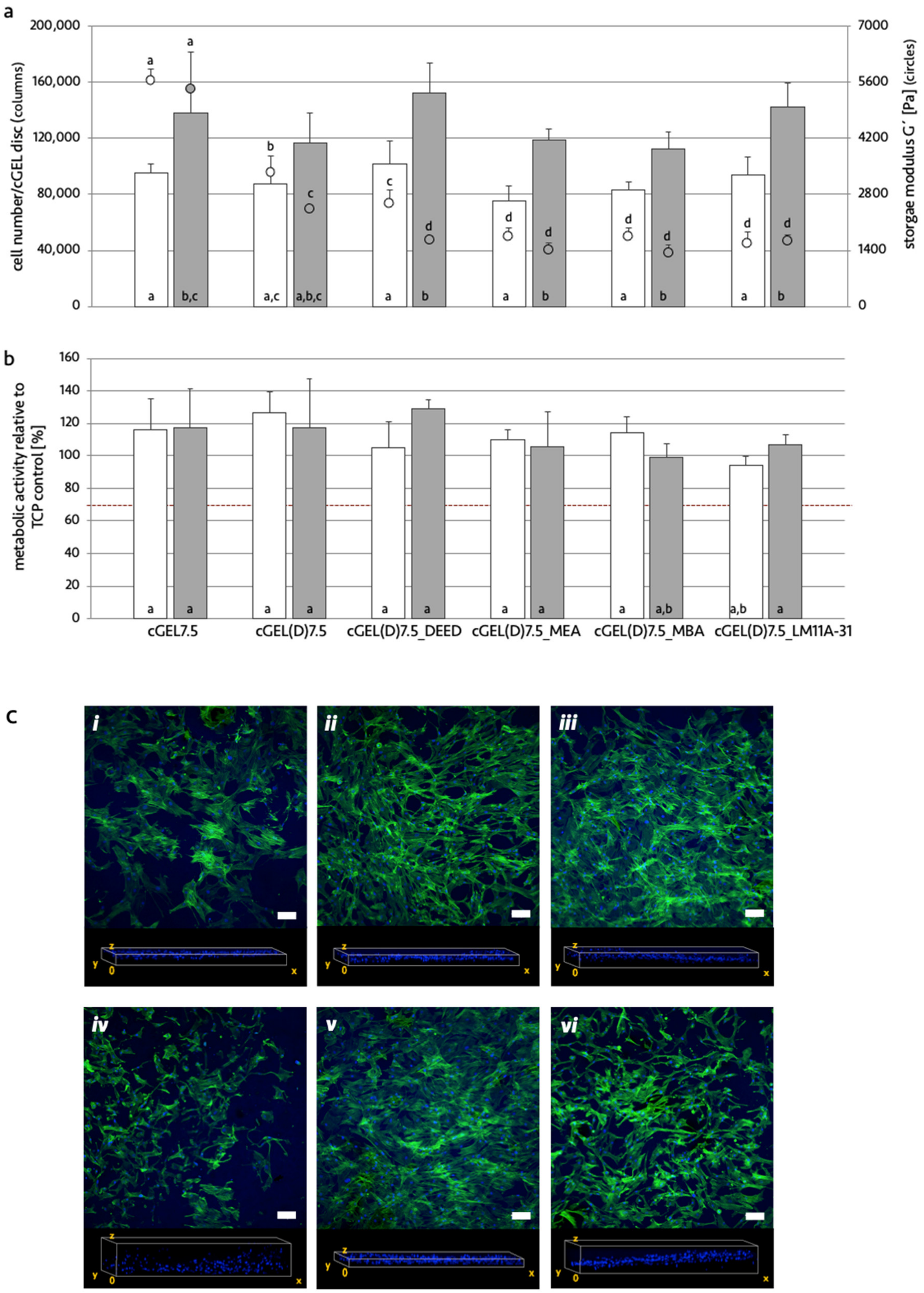

Figure S 2 Direct cell contact and cytocompatibility of cross-linked hydrogel matrices cGEL7.5, and pristine cGEL(D)7.5 that were based on prior cell proliferation studies. In this context, functionalized cGEL(D)7.5 matrices were included to evaluate the potential of amine integration on cytotoxicity, cell proliferation and cell spreading. a) Number of hASC on oPNMA-7.5and oPDMA.7.5 derived hydrogels (cGEL7.5 or cGEL(D)7.5 and amine modified cGEL(D)7.5_*amine* at day three (white columns) and day seven (grey columns), cell number quantified by Alamar Blue®. Storage moduli G´ of rehydrated

Figure S 2 continued.

cGEL discs in cell culture medium after day three (empty circles) and day seven (grey circle) incubation at 37 °C. Derivatization of oPDMA-7.5 to cGEL(D)7.5_*amine* with 2.5 anhydride equivalents (MAeq 2.5), small amines for derivatization: DEED: *N,N*-diethylethylendiamine, MEA: 4-(2-aminoethyl)morpholine, MBA: 4-(morpholin-4-yl)butan-1-amine, and LM11A-31: (2S,3S)-2-amino-3-methyl-*N*-[2-(4-morpholinyl)ethyl] pentanamide. b) Cytocompatibility of L929 mouse fibroblasts monitored via Alamar Blue®, cell viability measured after 24 h incubation with cGEL extracts (white columns) by fluorescent intensity upon resazurin reduction, measurement repeated after 48 h recovery of L929 fibroblasts in fresh cell culture medium (grey columns), L929 cellular response compared to positive control in standard tissue culture plate (TCP), dotted red lines represents 70% metabolic activity as cytocompatibility threshold. Columns and circles with error bars represent means + standard deviation (columns: n=4; circles: n=5). Means with different letters are statistically significantly different ($p<0.05$). c) Laser scanning microscopy images (z-stacks of at least 20 micrographs) of cGEL7.5 (*i*), cGEL(D)7.5 (*ii*), cGEL(D)7.5_DEED (*iii*), cGEL(D)7.5_MEA (*iv*), cGEL(D)7.5_MBA (*v*), cGEL(D)7.5_LM11A-31 (*iv*), cell staining on day seven is shown with Alexa Fluor® 488 phalloidin (green) and DAPI (blue); corresponding view of cell nuclei distribution in hydrogel frontal plane. Scale bars in z-stacks represent 100 μm.

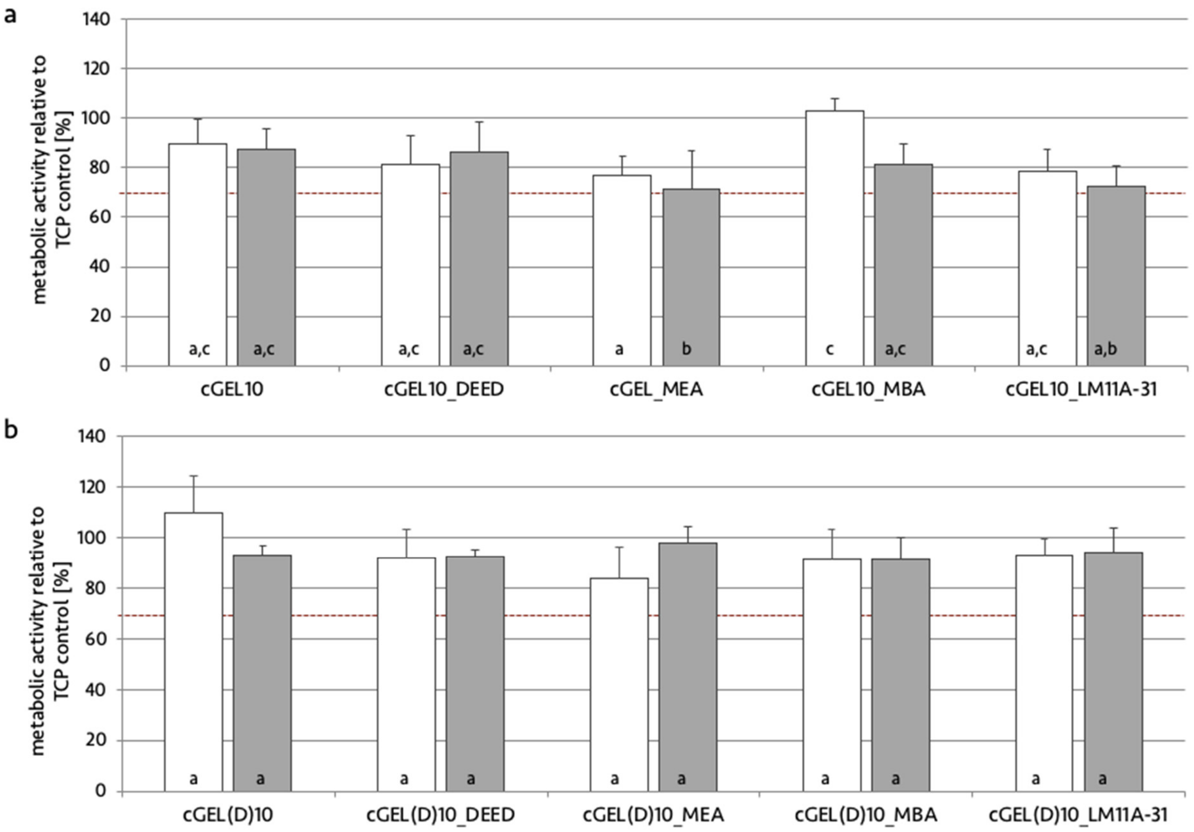

Figure S 3 Cytocompatibility of extracts derived from cGEL10 and cGEL(D) 10 to show cytocompatibility of the hydrogel material on L929 mouse fibroblasts. Extracts were taken from (a) cGEL10 and cGEL10_*amine* (samples as in Figure 5) and (b) cGEL(D) and cGEL(D)_*amine* (samples as in Figure 6) with DEED: *N,N*-diethylethylendiamine, MEA: 4-(2-aminoethyl)morpholine, MBA: 4-(morpholin-4-yl)butan-1-amine, and LM11A-31: (2S,3S)-2-amino-3-methyl-N-[2-(4-morpholinyl)ethyl] pentanamide). Cell viability of L929 fibroblasts monitored via Alamar Blue® assay after 24 h incubation with hydrogel extracts (white columns) and 48 h recovery in fresh cell culture medium (grey columns), L929 cellular response compared to positive control in standard tissue culture plate (TCP). Dotted red lines represent 70% metabolic activity as the cytocompatibility threshold. Columns with error bars represent means + standard deviation (n=4). Columns with different letters are statistically significantly different ($p<0.05$).

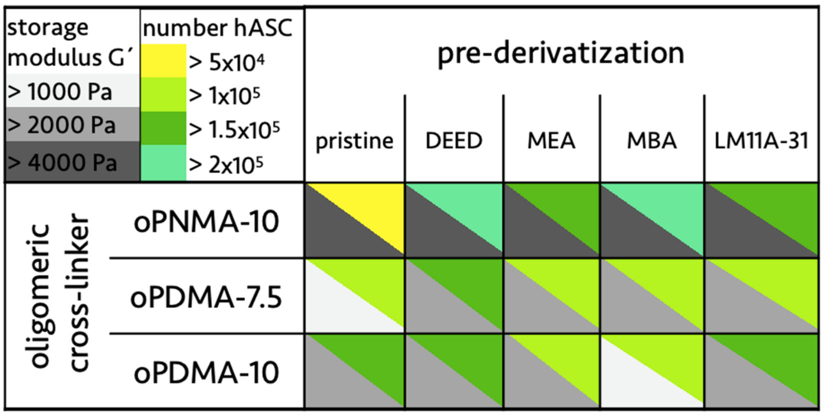

Figure S 4 Overview of hASC growth and hydrogel stiffness of pristine and derivatized matrices. Hydrogels derived from pristine and pre-derivatized oligomeric cross-linkers oPNMA-10, oPDMA-7.5 and oPDMA-10. Colours illustrate different ranges for mean values of storage modulus G´ (grey scale) and number adherent hASC at day 7 of date depicted in Figure 5, 6 and SI 2. Small amines for derivatization: DEED: N,N-diethylethylendiamine, MEA: 4-(2-aminoethyl)morpholine, MBA: 4-(morpholin-4-yl)butan-1-amine, and LM11A-31: (2S,3S)-2-amino-3-methyl-N-[2-(4-morpholinyl)ethyl] pentanamide.
